# Single and Multiple Gate Design Optimization Algorithm for Improving the Effectiveness of Fiber Reinforcement in the Thermoplastic Injection Molding Process

**DOI:** 10.3390/polym15143094

**Published:** 2023-07-19

**Authors:** Mattia Perin, Youngbin Lim, Guido A. Berti, Taeyong Lee, Kai Jin, Luca Quagliato

**Affiliations:** 1 Department of Management and Engineering, University of Padua, 36100 Vicenza, Italy; mattia.perin@phd.unipd.it (M.P.); guido.berti@unipd.it (G.A.B.); 2 SIMULIA, Dassault Systèmes Korea, Seoul 06164, Republic of Korea; lyb0684@naver.com; 3 Graduate Program in System Health Science and Engineering, Division of Mechanical and Biomedical Engineering, Ewha Womans University, Seoul 03760, Republic of Korea; tlee@ewha.ac.kr; 4 Division of Mechanical and Biomedical Engineering, Ewha Womans University, Seoul 03760, Republic of Korea; 5 School of Materials Science and Engineering, Ocean University of China, Qingdao 266100, China; jinkai@ouc.edu.cn

**Keywords:** injection molding, short fiber reinforced composite (SFRC), injection gate design, fiber orientation optimization, stiffness optimization, machine learning (ML), gradient boosting (GB)

## Abstract

Fiber reinforcement orientation in thermoplastic injection-molded components is both a strength as well as a weak point of this largely employed manufacturing process. Optimizing the fiber orientation distribution (FOD) considering the shape of the part and the applied loading conditions allows for enhancing the mechanical performances of the produced parts. Henceforth, this research proposes an algorithm to identify the best injection gate (IG) location/s starting from a 3D model and a user-defined load case. The procedure is composed of a first Visual Basic Architecture (VBA) code that automatically sets and runs Finite Volume Method (FVM) simulations to find the correlation between the fiber orientation tensor (FOT) and the IG locations considering single and multiple gates combinations up to three points. A second VBA code elaborates the results and builds a dataset considering the user-defined loading and constraint conditions, allowing the assignment of a score to each IG solution. Three geometrical components of increasing complexity were considered for a total of 1080 FVM simulations and a total computational time of ~390 h. The search for the best IG location has been further expanded by training a Machine Learning (ML) model based on the Gradient Boosting (GB) algorithm. The training database (DB) is based on FVM simulations and was expanded until a satisfactory prediction accuracy higher than 90% was achieved. The enhancement of the local FOD on the critical regions of three components was verified and showed an average improvement of 26.9% in the stiffness granted by a high directionality of the fibers along the load path. Finite element method (FEM) simulations and laboratory experiments on an industrial pump housing, injection-molded with a polyamide-66 reinforced with 30% of short glass fibers (PA66-30GF) material were also carried out to validate the FVM-FEM simulation frame and showed a 16.4% local stiffness improvement in comparison to the currently employed IG solution.

## 1. Introduction

Thermoplastic injection molding (TIM) is a widespread and well-established manufacturing process with several advantages, among them low cycle time, high dimensions accuracy and repeatability, low material waste, and recyclability of the material [1,2]. In recent years, scholars have addressed several weak points related to the TIM process, such as the defect detection strategies [3], warpage and shrinkage control [4], and weldline optimization to reduce their negative effect on the overall strength of the molded part [5].

Much effort has also been directed towards the inclusion of ever longer fibers, or fibers structures, such as woven reinforcements, within injection molded components. In this regard, in Jeong et al. [6], a strong improvement in terms of mechanical properties was achieved by processing a combination of a carbon fibers (CF) woven reticulated structure within a polypropylene (PP) matrix by the injection molding process. In addition, to comply with ever stricter regulations related to hydrocarbons, various researchers have also focused on how to process recycled reinforcements [7] in the TIM process and how to account for their variability during the part design stage [8]. Within this background, the design and manufacturing for the injection molding process is following the trend of other composite-related processes for lightweight design [9] and high-performance component manufacturing [10]. Another important aspect relevant to all fibers-reinforced composites (FRC), which also applies to the TIM process, is the influence of the fiber orientation distribution (FOD) on the mechanical performance of the component. As shown in recent literature studies, the FOD affects the static strength and fatigue life [11,12,13], the effect of the stress triaxiality and loading conditions on the failure behavior [14,15,16], and notch sensitivity [17,18].

Considering the strong influence on mechanical performance, the matter of predicting the FOD development within injection-molded components has attracted the attention of several scholars. From the analytical point of view, Huang and Zhao [19] proposed a generalized distribution function (GDF) for the FOD prediction based on the ratio between the shell and core layers developing throughout the thickness of injection molded components. The GDF formulation proved to be slightly superior (~5%) to the largely employed reduced strain closure (RSC) model proposed by Wang [20], normally coupled with the Anisotropic Rotary Diffusion (ARD) model [21], and successfully applied by Favaloro and Tucker [22] for the prediction of the FOD in the molding process of short FRC. The ARD-RSC model is easy to employ and calibrate and, for this reason, has been employed in various recent studies [11,14,17,23], and is also available within the Autodesk Moldflow Insight (AMI) 2023 environment [24,25,26], also employed in this research.

As concerns the TIM process, the FOD is mainly influenced by two factors, namely the part geometry and the injection gate location, or locations. For the case of unfilled polymers, the injection gate is normally designed to achieve a balanced polymer filling and avoid shortshots [27,28]. In recent years various gate location design algorithms have been proposed to minimize one or multiple issues related to the TIM process, such as the optimization/reduction of injection pressure, warpage, residual stresses, and weld lines [29,30]. In terms of FRC, Li et al. [31] proposed a methodology based on the backpropagation neural network and genetic algorithm–particle swarm optimization algorithm for the estimation of the influence of the main TIM process parameters on the FOD. Although interesting, the analysis considers a constant position for the injection gate and focuses only on the influence of the process parameters. Both aspects are crucial, but the latter can be adjusted during the initial pre-production batches, whereas a variation of the injection gate location is normally a highly time-consuming and expensive operation.

Considering the available literature, there seems to be a lack of knowledge in terms of the correlation between the injection gate location/s and the fiber orientation distribution arising within the component. Indeed, the geometry of the component plays an important role, but the FOD can be adjusted and designed by optimizing the location/s of the injection gates. To this aim, this research presents an injection gate (IG) design algorithm, based on a Visual Basic Architecture (VBA) script, able to automatically set and run FVM simulations, implemented in Autodesk Moldflow Insight 2023, and to automatically export the fiber orientation tensor (FOT) for each element of the FVM model mesh. At this point, the user is asked to define the regions of interest (RoI) in the model along with the relevant directions along which the FOT should be optimized. This phase can be carried out by preliminary finite element method (FEM) simulations or by previous analysis of the loading and boundary conditions applied to the component being investigated.

To limit the number of FVM simulations, the dataset composed of injection gate locations and element-based FOTs was employed for the training and validation of a machine learning (ML) model based on the Gradient Boosting (GB) method. The trained GB model is then employed to predict the FOT resulting from the application of IG in positions where no FVM simulations have been run, allowing for a full IG processing map, customized on the component being investigated. Machine learning approaches have been largely applied to other manufacturing processes [32,33] but the only application to the TIM process seems to be related to unfilled polypropylene [34,35] and targeted the end-product quality rather than its mechanical performances.

The accuracy of the proposed methodology, composed of the VBA interface algorithm, the FVM simulations, and the ML-GB model, was validated by considering three geometries with increasing complexity. In addition to that, to verify the effect of the optimized FOT on the mechanical response of the investigated component, the FVM models have been coupled with FEM simulations, implemented in Abaqus 2020, through the Autodesk Advanced Material Exchange (AME) 2019 platform, allowing us to account for the effect of the element-based FOT (FVM) on the element-based material properties (FEM).

From the FVM-FEM point of view, the proposed IG design algorithm allowed for a ~36% improvement in terms of FOT and a consequent increase in the global stiffness of 26.9%, respectively. To validate the whole FVM-FEM simulation framework, laboratory experiments have been carried out on an industrial pump housing manufacturing by TIM and by employing a polyamide-66 reinforced with 30% of short glass fibers (PA66-30GF) material. The results of the mechanical testing showed a negligible deviation between numerical and experimental load-displacement curves, while the local stiffness improved by 16.4% on average. This positive result proves that the FVM simulation is capable of accurately predicting the FOT and FOD and that the mapping operation (FVM → FEM) allows for a precise estimation of the global elastic response, calculated by the FEM simulations. Summarizing, the proposed approach has been developed considering single, double, and triple injection gate configurations, which also represent most of the cases from small to mid-size components manufactured by the TIM process. Moreover, thanks to its modularity, the algorithm can be extended to other FRC materials by recalibrating the orthotropic material properties in the FVM simulation.

## 2. Materials and Methods

This chapter is subdivided into 3 sections devoted to the following key aspects of this research. Section 2.1 details the material properties of the polyamide-66 reinforced with 30% of short glass fibers (PA66-30GF) material employed in all the finite volume method (FVM) simulations. This section also summarizes the process parameters and fiber orientation distribution (FOD) calculation model employed for the implementation of the FVM simulations. Section 2.2 presents the global working principle and the details for each phase for the proposed gate design algorithm and includes the details of the ML model based on the GB algorithm employed to expand the search for the best IG location. Finally, Section 2.3 summarizes the implementation strategy for the finite element method (FEM) simulations and the laboratory experiment procedures.

### 2.1. PA66-30GF Properties and FVM Simulation Settings

**Figure 1:**
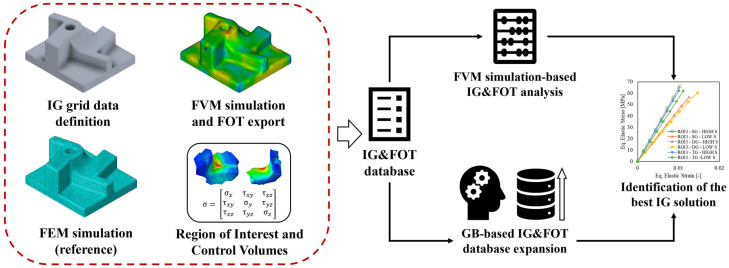
Material properties calibration and main phases of the FVM-FEM mapping.

**Figure 2:**
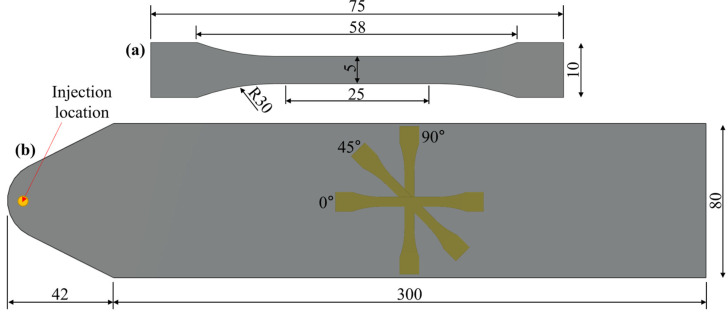
(**a**) BS-EN-ISO-5272012 type 1BA specimen’s dimensions and (**b**) 0° (injection direction), 45°, and 90° specimens’ machining positions on the injection molded plate (thickness: 2 mm).

**Figure 3:**
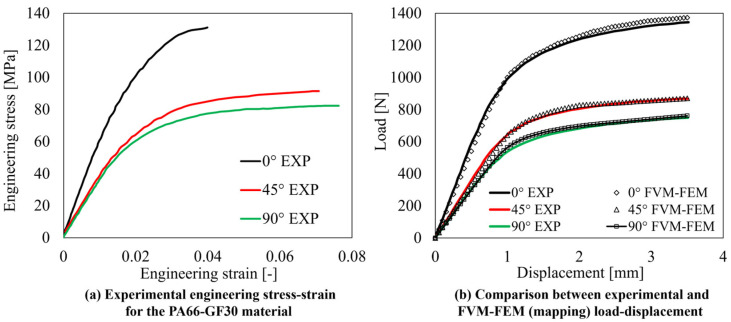
(**a**) Experimental engineering stress–strain curves for the PA66-30GF material [36] and (**b**) comparison between experimental and FVM-FEM numerical modeling for the load-displacement along the 0° (injection direction), 45°, 90° directions.

The material properties of the employed PA66-30GF material have been acquired from the work of Isaincu et al. [36] and inversely calibrated in the FVM simulation for further mapping into the FEM model. The rheological and thermal properties of the material, necessary for the implementation of the FVM simulation model, have been acquired from the Zytel 70G30HSL datasheet, a largely employed PA66-30GF material. The overall procedure encompassing the material properties calibration, the FVM simulation, the fiber orientation and material properties mapping, and the FEM simulation steps are reported in Figure 1. For the calibration of the material properties, BS-EN-ISO-5272012 type 1BA specimens, Figure 2a, have been machined from the central region of an injection molded plate (Figure 2b). This approach allows for the achievement of a uniform FOD in the calibrated region of the specimens, and thus for a precise estimation of the direction-dependent material properties. Specimens along 0°, 45°, and 90° have been considered, resulting in the engineering stress–strain curves of Figure 3a.

The material properties have then ben input into the Moldflow Insight environment as they are shown in Figure 3a and, by means of subsequent mapping calibration [11], the difference between experimental and numerical load-displacement curves have been minimized, as shown in Figure 3b. The material properties are interpolated considering the Ramberg–Osgood flow stress model, Equation (1), coupled with a modified Hill ’48 yield function, Equation (2), where *α* and *β* parameters refer to the fiber direction (0°) and transversal direction (90°), respectively, and scale the yield function account for the orthotropic nature of injection molded polymers.

(1) $$\sigma =E^{1/n}{\left(K\right)}^{\left(n-1\right)/n}{\left(\varepsilon_{p,eff}\right)}^{1/n}$$

(2) $$\sigma_{eff}=\sqrt{\frac{{\left(\alpha \sigma_{11}-\beta \sigma_{22}\right)}^{2}+{\left(\beta \sigma_{22}-\beta \sigma_{33}\right)}^{2}+{\left(\beta \sigma_{33}-\alpha \sigma_{11}\right)}^{2}+6\left[{\left(\sigma_{12}\right)}^{2}+{\left(\sigma_{23}\right)}^{2}+{\left(\sigma_{31}\right)}^{2}\right]}{2}}$$

Both *α* and *β* are calculated for each element of the FVM simulation mesh based on its relevant fiber orientation distribution, accounted for in terms of its first eigenvalue $\lambda_{I}$. The element-based calculation for both parameters is that of Equation (3) but refers to two different reference values, one for the injection direction (*α_m_*) and one for the transversal direction (*β_m_*). In Equation (3), $\lambda_{m,I}$ represents the first eigenvalue of the fiber orientation matrix in the region of the model with the highest fiber alignment with the polymer flow.

(3) $$\alpha \left(\lambda_{I}\right)=\theta +\left[\frac{\left(\alpha_{m}-\theta \right)}{\left(\lambda_{m,I}-1/2\right)}\right]\left(\lambda_{I}-1/2\right)\;,\;\beta \left(\lambda_{I}\right)=\theta +\left[\frac{\left(\beta_{m}-\theta \right)}{\left(\lambda_{m,I}-1/2\right)}\right]\left(\lambda_{I}-1/2\right)$$

The material constants relevant to Equations (1) and (3) are reported in Table 1 and define the full set of constants included in all FVM simulations for the estimation of the element-based material properties, based on the local fiber orientation, estimated through the ARD-RSC model [20,21,22], as hereafter reported.

As demonstrated in various recent works [11,14,17], the ARD-RSC model, Equation (4), is capable of accurately predicting the FOT during the thermoplastic injection molding (TIM) and carries two improvements in comparison to the Folgar–Tucker model [37].

(4) $$\begin{array}{l} \frac{\partial a_{\mathrm{ij}}}{\partial t}=-\frac{1}{2}\left(w_{\mathrm{ik}}a_{\mathrm{kj}}-a_{\mathrm{ik}}w_{\mathrm{kj}}\right)+\frac{1}{2}\lambda \left({\dot{\gamma }}_{\mathrm{ik}}a_{\mathrm{kj}}+a_{\mathrm{ik}}{\dot{\gamma }}_{\mathrm{kj}}-2{\dot{\gamma }}_{\mathrm{kl}}\left[a_{\mathrm{ijkl}}+\left(1-k\right)\left(L_{\mathrm{ijkl}}-M_{\mathrm{ijkl}}\cdot a_{\mathrm{mnkl}}\right)\right]\right)\\ +{\dot{\gamma }}_{\mathrm{ij}}\left[2\left(c_{\mathrm{ij}}-\left(1-k\right)c_{\mathrm{kl}}M_{\mathrm{ijkl}}\right)-2k\cdot c_{\mathrm{kk}}a_{\mathrm{ij}}-5\left(c_{\mathrm{ik}}a_{\mathrm{kj}}+a_{\mathrm{ik}}c_{\mathrm{kj}}\right)+10c_{\mathrm{kj}}\left(a_{\mathrm{ijkl}}+\left(1-k\right)\left(L_{\mathrm{ijkl}}-M_{\mathrm{ijkl}}\cdot a_{\mathrm{mnkl}}\right)\right)\right] \end{array}$$

**Figure 4:**
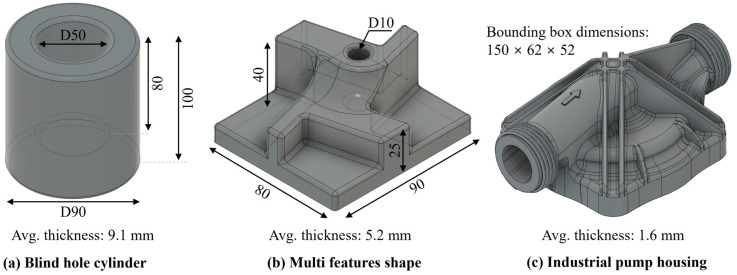
Training geometries with increasing complexity as considered in this research. (**a**) Blind hole cylinder, (**b**) multi features shape, and (**c**) industrial pump housing. The arithmetic average thickness is calculated as the ratio between the volume and external area of the part.

The former is related to the fiber-to-fiber interaction within the polymeric flow, thus occurs during the injection phase of the process. This is carried out by the fiber interaction function ($c_{\mathrm{ij}}$), which substitutes the fiber interaction constant of the Folgar–Tucker model. In this scenario, $c_{\mathrm{ij}}$ represents a quadratic function calculated on the basis of the fiber orientation tensor ($a_{\mathrm{ij}}$) and of the deformation rate tensor (${\dot{\gamma }}_{\mathrm{ij}}$). In addition to that, the reorientation of the fibers during the injection phase is controlled by an improved closure term identified by $\left[a_{\mathrm{ijkl}}+\left(1-k\right)\left(L_{\mathrm{ijkl}}-M_{\mathrm{ijkl}}\cdot a_{\mathrm{mnkl}}\right)\right]$, where $L_{\mathrm{ijkl}}$ and $M_{\mathrm{ijkl}}$ tensors are calculated as the products between eigenvalues and eigenvectors components of the orientation tensor ($a_{\mathrm{ij}}$). The fiber reorientation constant allows for scaling between a fully allowed reorientation during the injection phase (k ≈ 1) to a highly limited reorientation (k ≈ 0). In this research, the reorientation constant has been calibrated to k = 0.6, by employing the same procedure defined and employed in Quagliato et al. [14]. By considering this modeling background, all FVM simulations related to this research have been implemented in the Autodesk Insight 2023 environment considering a tetrahedral mesh where the element side length and aspect ratio have been set to 3 mm and 4 mm, respectively. The three geometries, employed for training and validation purposes, are reported in Figure 4 along with their relevant main dimensions. Given that the injection molded industrial pump housing of Figure 4c is related to a commercial product, only a few dimensions details can be disclosed.

In all FVM simulations, the injection gate (IG) has been modeled without the relevant injection channels and runners. Although injection channels and runners might have an influence on the FOT, especially for complex geometries, in this study, they have been neglected to reduce the modeling and computational effort. In terms of process parameters, for the injected plate of Figure 2 and the three components of Figure 4, the details are reported in Table 2 and are related to the injection phase, post-pressure, and cooling phase. Since this research aims at the investigation of the influence of injection gate location, fiber orientation distribution, and component stiffness, the warpage calculation phase was omitted from the FVM simulations. In Table 2 the velocity/pressure switchover refers to the part volume filling percentage whereas the post-pressure refers to the percentage with respect to the maximum pressure during the filling phase.

### 2.2. Single and Multiple Injection Gate Design Algorithm

**Figure 5:**
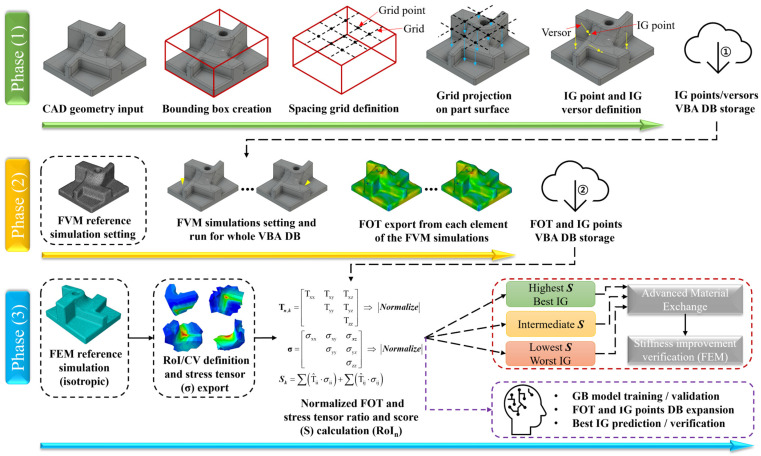
Main phases and tasks of the injection gate design algorithm.

The injection gate (IG) design algorithm is composed of three main sections, as summarized in Figure 5. The first phase (1), based on an Ilogic script implemented in Autodesk Inventor 2023, handles the CAD model and is responsible for the definition of all the possible injection locations on the considered geometry. The locations are defined considering a user-defined spacing strategy, in which the bounding box enveloping the component is used to project the IG locations on the inner and outer surfaces of the CAD geometry.

The grid spacing employed for all the considered components of Figure 4 is, in general, equal to 15 mm and is the result of a grid size optimization considering 10 mm, 15 mm, and 20 mm, carried out before the beginning of phase (1). Although highly time-consuming, if the grid size is intended to be further optimized, the whole procedure of Figure 5 can be repeated considering different grid size values until convergency is reached. The IG candidate locations are defined by an (x,y,z) vector on the model and by the orthogonal versor **v** to the part surface (Figure 5). Using this approach, the defined IG candidates are constrained from belonging to inner or inaccessible surfaces and, once the IG candidates set is defined, it is automatically stored in an Excel VBA database (IG points/versors VBA DB storage ①).

Afterward, in phase (2), a second VBA script automatically creates all the FVM simulation input files and runs them automatically. All FVM simulations are based on the same mesh and process conditions, defined in a reference FVM simulation, which must be manually defined by the user. To this aim, as also carried out in this research, the process conditions and mesh should be checked a priori. In principle, there are no limitations to the number of FVM simulation cases within the same Autodesk Moldflow Insight (AMI) file, but for practical file handling reasons, the number of cases for a single simulation file is constrained to 350. Thus, if the IG test points are more than 350, more than one AMI simulation file is created. After all the FVM simulations relevant to the IG test points have been completed, a results export macro, directly implemented in the AMI environment, exports the fiber orientation tensor for each element of the mesh and combines them with the relevant IG coordinates (FOT and IG points VBA DB storage ②), as shown in Figure 5.

At this point, preliminary considerations can be carried out considering the relationship established between the IG locations and the relevant FOT on the critical regions of the component. However, to create a direct correlation between IG locations and the mechanical response of the component, two additional steps for phase (3) are required.

First, an FEM simulation with isotropic material properties is set considering the loading and boundary conditions (BC) applied to the component being designed and, in this research, it is employed to define the critical regions of the model. By considering isotropic material properties in the reference FEM simulation, the load path developing within the component depends only on the geometry and on the applied loading and boundary conditions. At this point, the user is requested to define the number of regions of interest (RoI) on the model and the relevant control volume (CV). The RoI are defined as positions in the considered geometry where the design engineer is interested in optimizing the FOD to achieve a better reinforcement efficiency. On the other hand, the CV is defined in terms of spherical volume within which the stress tensor components are exported and averaged after the completion of each FEM simulation. In the developed algorithm each RoI is associated with a customizable CV, set here to a 5 mm radius, allowing for the estimation of the influence of the IG location/s on the elastic response of the component.

After these two preliminary steps of phase (3), the normalized FOT and the normalized stress tensor estimated from the reference FEM simulation are calculated. In Equation (5), the FOT tensor ($T_{n,k}$) is estimated for each of the *n*-th FVM simulations, for every *k*-th RoI, and by considering the previously defined CVs.

(5) $$\begin{array}{l} T_{n,k}=\left[\begin{array}{ccc} T_{\mathrm{xx}} & T_{\mathrm{xy}} & T_{\mathrm{xz}}\\ & T_{\mathrm{yy}} & T_{\mathrm{yz}}\\ & & T_{\mathrm{zz}} \end{array}\right]\;\Rightarrow \;\left|Normalize\right|\;\Rightarrow \;{\hat{T}}_{n,k}\left[0\;\sim \;1\right]\\ \sigma =\left[\begin{array}{ccc} \sigma_{\mathrm{xx}} & \sigma_{\mathrm{xy}} & \sigma_{\mathrm{xz}}\\ & \sigma_{\mathrm{yy}} & \sigma_{\mathrm{yz}}\\ & & \sigma_{\mathrm{zz}} \end{array}\right]\;\Rightarrow \left|Normalize\right|\Rightarrow \;\hat{\sigma }\left[0\;\sim \;1\right] \end{array}$$

Moreover, both FOT and stress tensor (σ) are considered positive values since the directionality of the fibers is considered beneficial in the same way both for tensile and compressive stress states. Afterward, considering the *k*-th RoI in the *n*-th FVM simulation, the relevant score is calculated as in Equation (6) by separately considering the normal and shear stress components. The global IG score for the *n*-th FVM simulation is then estimated by a weighted average of all the *k*-th scores from each RoI according to Equation (7). The weights associated with each RoI ($\delta_{k}$) allow the designer to assign a higher or lower priority to different regions of the model while accounting for all of them at the same time.

(6) $$S_{k}=\sum \left({\hat{T}}_{\mathrm{ii}}\cdot {\hat{\sigma }}_{\mathrm{ii}}\right)+\sum \left({\hat{T}}_{\mathrm{ij}}\cdot {\hat{\sigma }}_{\mathrm{ij}}\right)\;\left[0\leq S_{k}\leq 1\right]$$

(7) $$S_{n}=\frac{1}{K}\sum_{k=1}^{K}S_{k}\cdot \delta_{k}\;\left[0\leq \delta_{k}\leq 1\right]$$

By repeating this procedure for all the *N*-FVM simulations for the considered component the weighted scores considering all the RoI are estimated, and the best and worst IG locations for the considered loading and boundary conditions can be defined, as shown in Figure 5. To verify the stiffness improvement, the FVM simulation results are mapped through the AME module to the Abaqus 2020 FEM simulation model.

Since the geometry, loading, and boundary conditions are the same, the stiffness variation predicted by the FEM simulations is influenced only by the FOT, which is in turn influenced by the IG gate location/s. After the normalization and scores calculation, the user has also the option of expanding the search for the optimal IG by employing a machine learning (ML) Gradient Boosting model (GB), as defined and employed in Mirandola et al. [32], Equation (8).

(8) $$\Upsilon (w)=L_{\delta }(y,f)=\left\{ \begin{array}{ll} \frac{1}{2}{\left(y-f\right)}^{2} & \mathrm{if}\;\;\left|y-f\right|<\delta \\ \delta \left|y-f\right|-\frac{1}{2}\delta^{2} & \mathrm{otherwise} \end{array}\right.$$

In Equation (8), $L_{\delta }$ is defined as the loss function and is employed to update the prediction between the *m-step* and the *m* + 1 *step*. The new prediction is based on the difference between predicted and true values between two consecutive iterations. In the GB algorithm, only one tree (f) is built and progressively updated according to Equation (9), where η is the learning rate and defines the speed at which the loss function is minimized.

(9) $$f_{m+1}=f_{m}+\eta \cdot r_{m+1}\;\mathrm{where}\;\;r_{i,m+1}=-{\left|\frac{\partial L_{\delta }(y_{i},f_{i})}{\partial f_{i}}\right|}_{f_{m}}$$

The GB model is trained on the basis of the FVM simulation results, in terms of IG (x,y,z) coordinates and $S_{k}$ scores. The objective function is defined in terms of coordinates of the IG gate, or gates, that allow for the maximization of the $S_{k}$ scores or the weighted $S_{n}$ score. To assess the accuracy of the training, a 5-fold validation is automatically carried out after the training, allowing the user to decide whether to include additional FVM simulation and repeat the training process or accept the proposed solution. After the training process, the GB algorithm is requested to predict either the $S_{k}$ scores or the $S_{n}$ score on a user-defined and finer grid than that employed in phase (1), Figure 5. This approach allows for more accurate tuning of the IG location design while also granting the estimation of a feasible solution. The consideration of a post-processing phase based on the GB algorithm allows for finer tuning of the IG location, thus for a better improvement of the component stiffness, especially for the case of complex geometries.

The Ilogic script (Autodesk Inventor 2023), the two VBA scripts for phases (1) and (2), the normalization VBA script for phase (3), the GB Phyton script, and the output DB of phase (2), FOT and IG points VBA DB, are available as Data Availability Statement to this paper, stored on an online repository, or on request to the corresponding author.

### 2.3. FEM Simulation Settings and Validation Experiments

**Figure 6:**
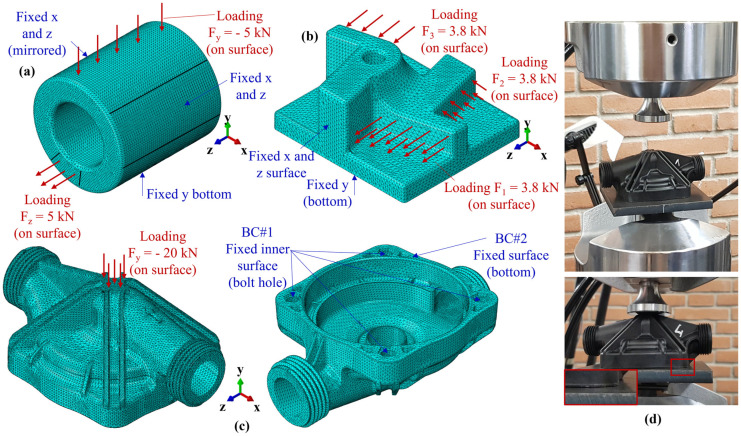
Mesh, loading, and boundary conditions for the (**a**) Blind hole cylinder, (**b**) Multi features shape, and (**c**) Industrial pump housing. (**d**) Experimental setup for the industrial pump housing.

The FEM simulations, partially introduced in the previous section of the paper, have been implemented in Abaqus 2020 considering a static/implicit solution scheme. All three models of Figure 4 have been meshed considering a C3D10H element with a general size of 2 mm and refinement of 0.8 mm, for a total of 442,639 (a), 130,051 (b), and 374,401 (c) elements, respectively. The meshes have been tested considering two coarser and one finer mesh approaches, allowing us to conclude that the employed strategy is the best tradeoff between accuracy and computational time. For all meshes, a quadratic integration scheme has been employed to allow a better representation of the stress distribution within the element, thus catching the complex behavior related to an element-based material properties mapping. The loading and boundary conditions applied to the models are reported in Figure 6a–c. For the setting of the reference FEM models needed for phase (3) of Figure 5, isotropic material properties have been considered as E = 14 GPa and ν = 0.37. Since the aim of the reference FEM simulation is only the identification of the load path, in terms of RoI and directions of maximum stress, the selected material properties do not affect the score calculation, as presented in the previous section of the paper.

As concerns the validation experiments, the testing conditions of Figure 6d were employed together with the same BC for the bottom of the part employed in the relevant FEM simulation, as shown in Figure 6c. Moreover, since this research focuses only on the elastic response, the experiments have been carried out right across the yield point and then stopped before severe deformation arose on the component. For the experiments, the MTS 809 axial/torsional test machine was employed where the reaction force was measured by the embedded load cell and the vertical displacement by the machine encoder. A total of 5 pieces were tested with a 2 mm/min downward speed, and the relevant load-displacement curves, together with the FVM-FEM estimation, are reported in Section 3.3.

## 3. Results

According to the research implementation frame presented in Section 2 of the paper, this section is organized in a similar manner to provide a one-to-one connection with the relevant methodological background.

### 3.1. IG Solutions and RoI Scores Calculation

**Figure 7:**
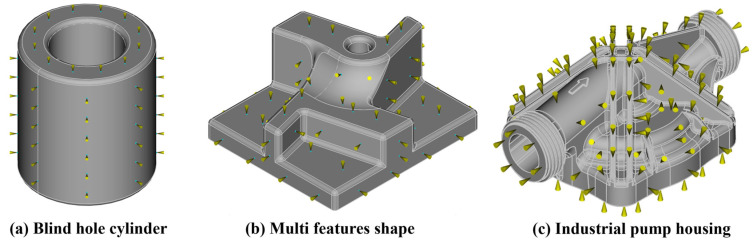
Examples of IGs generated on the user-defined grid on (**a**) blind hole cylinder, (**b**) multi features shape, and (**c**) industrial pump housing.

Considering the automatic IG generation algorithm from Section 2.2, an example of the IG grid on the three components of Figure 4 is displayed in Figure 7. In the FVM simulation environment, the IGs are considered one by one, in the case of a single injection gate (IG = 1), in couples, in the case of a double injection gate (IG = 2), or in triplets, in the case of a triple injection gate (IG = 3). For the case of a single gate, each one of the IGs represents a single FVM simulation, whereas for IG = 2 and IG = 3, the combinations between the different locations are randomly generated to assure the absence of biases, especially for the training and validation of the GB-ML model, as presented in following Section 3.4.

**Figure 8:**
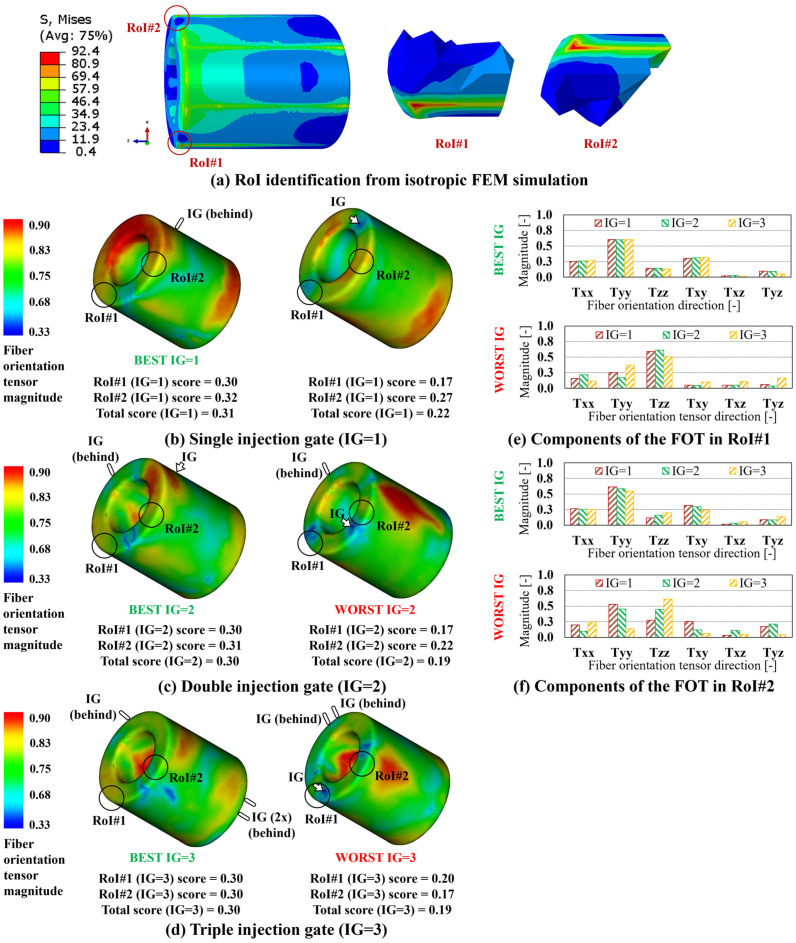
Blind hole cylinder results. (**a**) Von Mises stress distribution and RoI identification from isotropic FEM simulation. FOD on best and worst IG location for (**b**) IG = 1, (**c**) IG = 2, and (**d**) IG = 3. FOT components on the best and worst IG location for IG = 1, 2, 3 for (**e**) RoI#1 and (**f**) RoI#2.

Starting with the simplest geometry, the blind hole cylinder of Figure 4a, the application of bending and tensile loads, coupled with isotropic material properties, results in the von Mises equivalent stress distribution reported in Figure 8a, allowing for the identification of two RoIs. As previously mentioned, this preliminary FEM simulation aims to identify the regions of the model where the FOD ought to be aligned with the load path to improve the local stiffness. Accordingly, FVM simulations considering IG = 1, 2, 3 have been implemented and their total number, for all three geometries, is summarized in Table 3. The number of simulations reported in Table 3 has been progressively increased to achieve a sufficient training dataset for the GB-ML model of Section 3.4.

**Figure 9:**
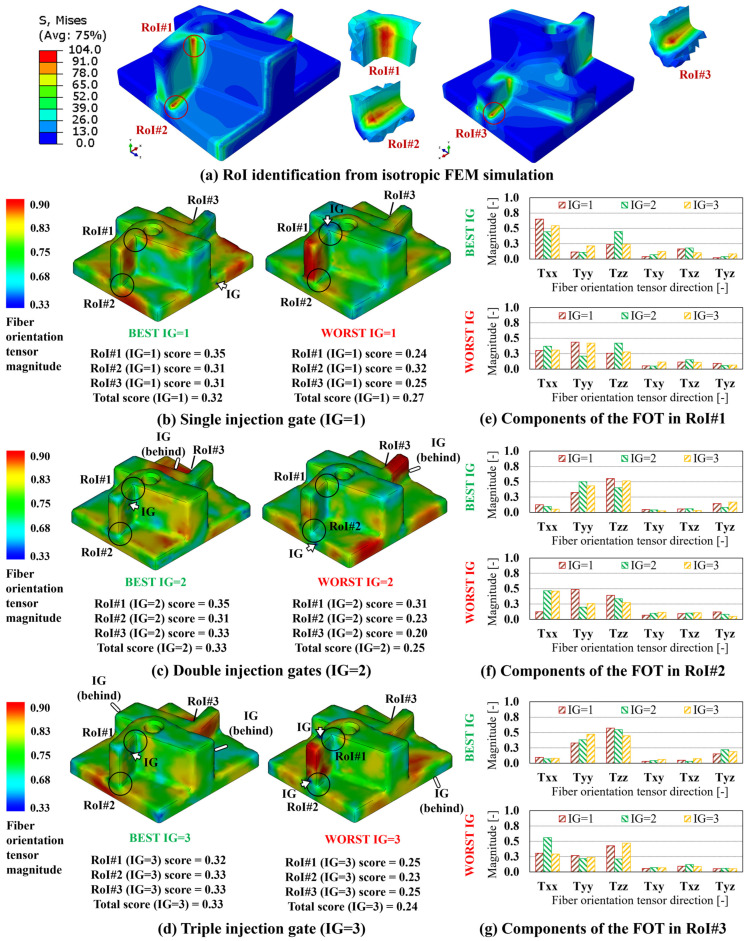
Multi features shape results. (**a**) Von Mises stress distribution and RoI identification from isotropic FEM simulation. FOD on best and worst IG location for (**b**) IG = 1, (**c**) IG = 2, and (**d**) IG = 3. FOT components on the best and worst IG location for IG = 1, 2, 3 for (**e**) RoI#1, (**f**) RoI#2, and (**g**) RoI#3.

**Figure 10:**
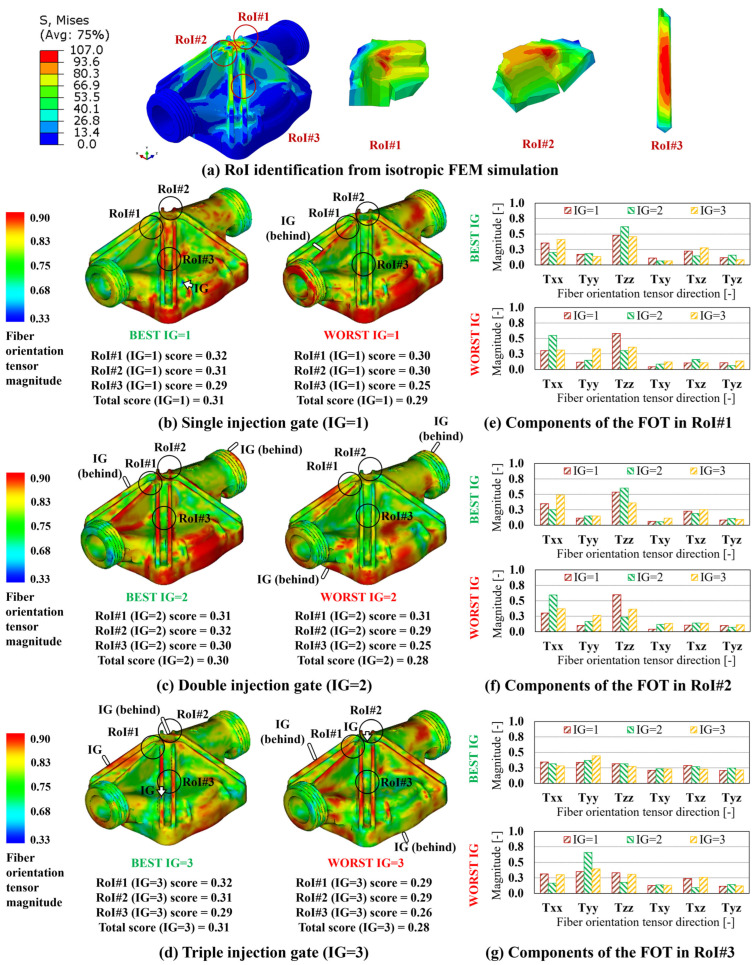
Industrial pump housing results. (**a**) Von Mises stress distribution and RoI identification from isotropic FEM simulation. FOD on best and worst IG location for (**b**) IG = 1, (**c**) IG = 2, and (**d**) IG = 3. FOT components on the best and worst IG location for IG = 1, 2, 3 for (**e**) RoI#1, (**f**) RoI#2, and (**g**) RoI#3.

For each FVM simulation, and each RoI, the scores have first been calculated, according to Equation (6), allowing for the determination of the total score for each case, Equation (7), and for the definition of the best and worst IG configuration, for single, double, and triple injection gates. In Figure 8b–d, the FOD resulting on the blind hole cylinder component when the best and worst IG configurations, for IG = 1, 2, 3, are considered and reported, along with the relevant RoI-based and total scores. In addition, in Figure 8e,f, the FOT components, for IG = 1, 2, 3, for the best and worst IG configurations, are reported for RoI#1 and RoI#2. Following the same strategy, the summary of the results relevant to the multi features shape and industrial pump housing are reported in Figure 9 and Figure 10, respectively. In the case of these two components, since three RoIs are considered, three comparison charts reporting the components of the FOD are reported.

Considering altogether the results relevant for the variation of the FOD between the best and worst IG location, the improvement in directionally of the FOT, calculated between the worst and best total scores among those reported in Figure 8, Figure 9 and Figure 10, is estimated at 63.2% for the blind hole cylinder, 35.7% for the multi feature shape, and 10.7% for the industrial pump housing, respectively.

At this point, to verify the stiffness improvement granted by the higher directionally of the fiber reinforcement, FEM simulations have been implemented considering the local mechanical properties by mapping them onto the structural simulation mesh by means of the Autodesk AME interface [11,14], as reported in the following section of the paper.

### 3.2. FVM-FEM-Based IG Design and Stiffness Improvement

Considering the same rationale employed in Section 3.1, the results relevant to the structural FEM simulations for the worst and best IG locations identified from the results of the FVM simulations, as reported in Section 3.1, are summarized in this chapter.

**Figure 11:**
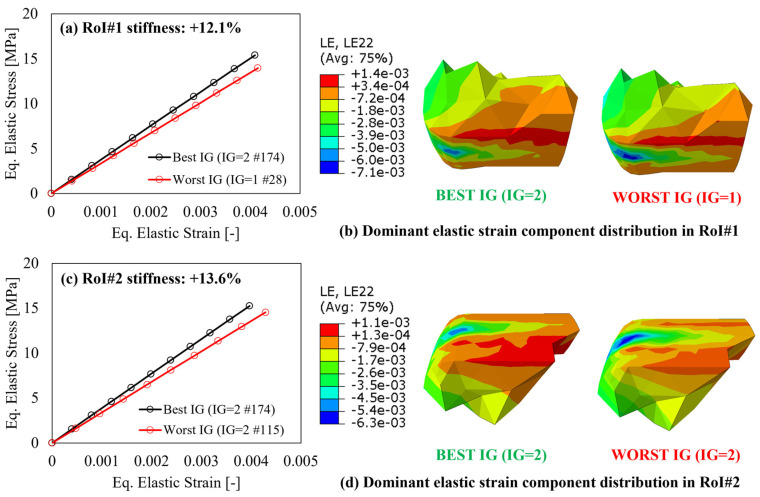
After-mapping local stiffness variation and major elastic strain component distribution for the blind hole cylinder in (**a**,**b**) RoI#1 and (**c**,**d**) RoI#2.

**Figure 12:**
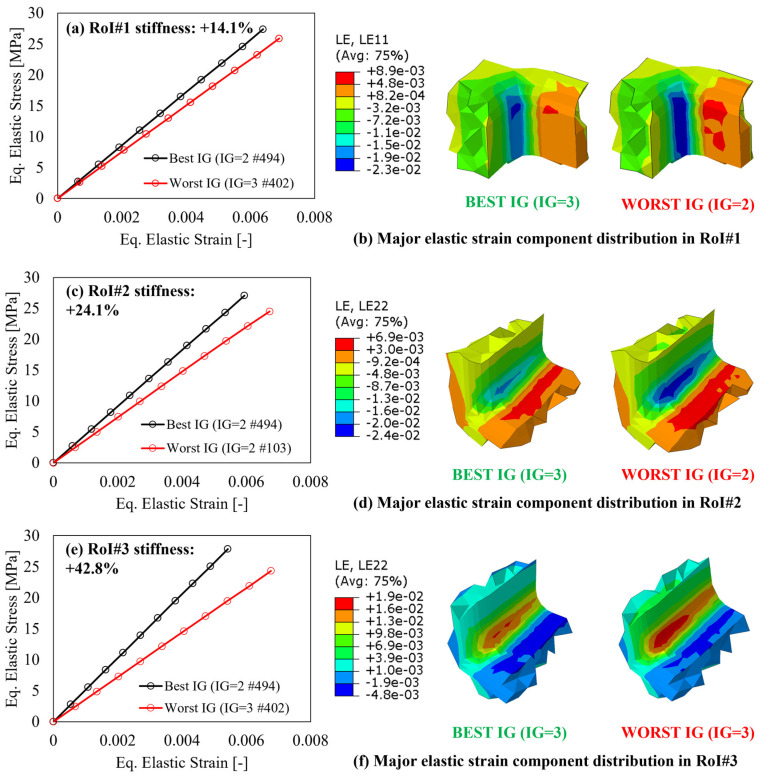
After-mapping local stiffness variation and major elastic strain component distribution for the multi features shape in (**a**,**b**) RoI#1, (**c**,**d**) RoI#2, and RoI#3 (**e**,**f**).

**Figure 13:**
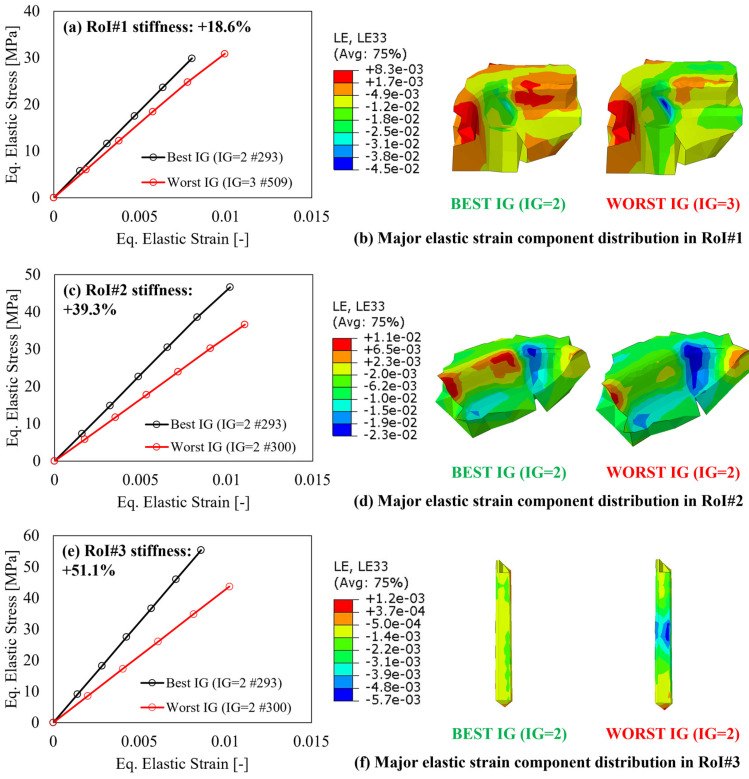
After-mapping local stiffness variation and major elastic strain component distribution for the industrial pump housing in (**a**,**b**) RoI#1, (**c**,**d**) RoI#2, and RoI#3 (**e**,**f**).

First, to verify the variation of the elastic response in the RoI, as a consequence of the applied external loads and boundary conditions, the equivalent stress–strain curves calculated according to the von Mises criterion, averaged within each CV relevant for each RoI, for three parts, are reported in Figure 11, Figure 12 and Figure 13, respectively. In all charts, the increase in the local stiffness granted by the best IG location, compared to the worst one, shows the benefit provided by the proposed IG design algorithm and is only related to the number and locations of the IG and no modifications of the components’ geometry.

Considering the blind hole cylinder, seen in Figure 11, the variation between the best and worst IG locations results in a 12.1% stiffness improvement in RoI#1 (Figure 11a) and 13.6% in RoI#2 (Figure 11c), respectively. In addition, as shown in the images comparison of Figure 11b,d, the variation of the IG location from worst to best allows for a reduction in the strain component along the main direction of the load path, namely the y-direction (LE22) for both RoI#1 and RoI#2.

As concerns the multi features shape, the results are summarized in Figure 12 following the same approach as Figure 11 but considering the three RoIs identified in Figure 9. For the case of this component, it is interesting to underline that the load path direction is not the same for the three RoIs. In fact, both RoI#2 and RoI#3 show the highest deformation along the y-direction, thus the relevant images show the elastic strain distribution along this direction (LE22). On the other hand, for RoI#1, the load path is most critical along the x-direction (LE11). Regardless of the direction along which the load path creates the highest deformation, the best IG, identified in double gate #494, allows for a reduction in the equivalent strain for the same equivalent stress, resulting in an average increase in the elastic modulus of 27%. In addition, it must also be considered that the mentioned reduction of the equivalent elastic strain does not necessarily refer to a tensile strain, but to a reduction in the magnitude of the strain component along the considered major direction.

The results relevant to the industrial pump housing, seen in Figure 13, as those for the multi features shape, offer another interesting insight into the possible conflict that might arise when optimizing multiple RoIs on the same component and at the same time. Regarding the first RoI, located in the proximity of the load application area, the improvement granted by the proposed IG design algorithm is equal to 18.6% but is rather lower than that on the other two RoIs. In fact, RoI#2 and RoI#3 are located on rib regions and show a far higher stiffness improvement, quantified in 39.3% and 51.1%, respectively.

Regardless of the IG configuration, when an RoI is selected close to a load application area, the non-uniformity of the load path results in a multiaxial stress state, which makes the optimization of the fiber orientation complex to achieve. Nevertheless, when the stress state becomes close to a hydrostatic, a random fiber orientation results in a better response of the material since no clear uniaxial load path can be identified.

On the other hand, for the case of RoI#2 and RoI#3, the load path follows the geometrical shape of the rib, with a consequent higher benefit from the alignment of the reinforcement towards the relevant direction. Considering the results of Figure 13, the following section shall provide a validation of the FEM and FVM simulation framework as well as the improvement granted by the proposed IG design with respect to that currently employed for the industrial pump housing.

### 3.3. FVM/FEM Validation and Benefits of IG Design Improvement

**Figure 14:**
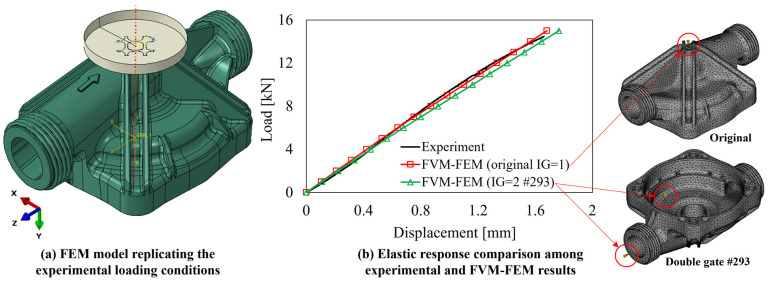
(**a**) FEM model implementation replicating the experimental conditions with the addition of the pressure plate on the top and (**b**) load-displacement curve comparison including the experimental, original IG FVM-FEM, and optimized IG configurations (double gate #293).

To evaluate the accuracy of the implemented FVM and FEM numerical simulation frame, the FOD resulting from the FVM simulation considering the original IG configuration and that of the best IG reported in Figure 13 have been mapped onto the FEM simulation model, replicating the loading conditions reported in Figure 6d. For this purpose, the load has been applied considering a flat punch, defined as analytically rigid, where the same 20 kN load conditions of Figure 6c were applied, as shown in Figure 14a. This modeling approach allows a closer replication of the experimental loading conditions as well as an easier export of the results from the reference point (RF) located on the rigid geometry. As shown in Figure 14b, the FVM-FEM simulation replicating the original IG condition well matches the experimental curve until it starts to bend due to the beginning of the yield phase in the material. In addition, the variation in the rigidity of the parts resulting from the variation of the IG (original → IG = 2 #293), estimated in terms of the ratio between load and displacement, shows a reduction of approximately 5.4%, which is fairly negligible and comparable to calculation errors in both FVM and FEM simulation models.

**Figure 15:**
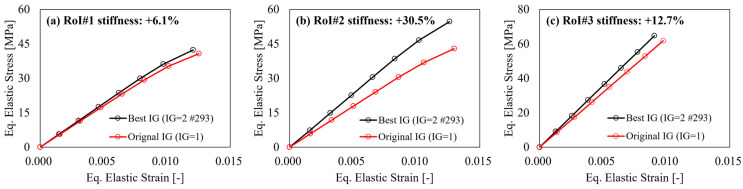
Equivalent stress–strain curves showing the stiffness variation in the pump housing between the original and double gate #293 IG solutions for (**a**) RoI#1, (**b**) RoI#2, and (**c**) ROI#3.

As concerns the stiffness improvement on the three RoIs identified for the industrial pump housing, the comparison between the equivalent elastic stress–strain curves, with respect to the original IG configuration (Figure 14b), is reported in Figure 15. For the case of RoI#1, the proximity with the load application area makes it complicated to improve the FOD effectiveness in this area, as also testified by the similarity in stiffness response between the original and double gate #293 IG = 2 solutions (Figure 15a).

On the other hand, for RoI#2 and RoI#3, the improvements are quantified in 30.5% and 12.7%, respectively, and show the improvement of the predicted IG design with respect to that currently employed solution. In addition to that, the stiffness improvements for all three RoIs are within the ranges previously presented in Figure 13, proving that the implemented algorithm can, in fact, define the upper and lower boundaries of the fiber alignment capabilities, and thus stiffness improvement, in the considered regions of the model.

### 3.4. Gradient Boosting-Based IG Design Optimization

This last section aims to provide insights on the additional improvements obtainable by considering the post-processing optimization where the results of the FVM simulations, as reported in Section 3.1, are used as a training dataset for the GB-ML model presented in Section 2.2. The training dataset is composed of the coordinates of the IG and the scores for each RoI identified from the results of the FVM simulation with respect to the preliminary FEM model. The target of this ML-based post-processing optimization is the identification of possible alternative IG locations that may allow for further improvement and that were not included in the initial grid, as shown in phase (1) of Figure 5.

First, the training and validation of the GB model have been carried out for the three considered geometries, thus the hyperparameters have been calibrated separately based on the GridSearch algorithm, as reported in Table 4. In the same table, the average deviations, calculated in terms of accuracy between the true and predicted score for each IG, are also reported and are the average of the 5-fold validation process. During the training and validation process, 80% of the dataset has been employed for training, whereas the remaining 20% is for validation.

Considering the trained GB-ML model, a search on a 5 mm grid was carried out on the whole inner and outer surfaces of the three geometries. The results, reported in Table 5, show the GB-predicted IG locations and the comparison with those previously estimated through the FVM-FEM modeling process. The notations IG = 2(#1) and IG = 2(#2) refer to the first and second IG locations in the considered best double gate configuration. The Pre GB-ML results are those relevant for the IG locations predicted only considering the results of the FVM simulations, whereas the Post GB-ML are those predicted by the trained GB model by adding virtual results on a finer grid size. Considering the Post GB-ML in Table 5, each ML prediction for the three best IG configurations has been further investigated by an FVM simulation, subsequent mapping, and FEM simulation, allowing us to verify the correctness of the predicted IG solution. The stiffness improvement results of Table 5 refer to the best IG reported in Figure 11, Figure 12 and Figure 13.

As shown in Table 5, the predictions obtained by the trained GB model are not always similar to those previously predicted by the FVM-FEM simulations (Table 5 and Figure 11, Figure 12 and Figure 13) but nevertheless allow for the plastic flow to obtain an FOD distribution aligned with the load path in the considered RoI. This fact shows that the best IG configuration is not univocal but, instead, more than one best IG candidate with similar potential can be identified within the same component and for the same load/constraint conditions. On the other hand, the ML predictions confirmed that the best IG solutions for all three components are represented by an IG = 2, as also shown in Figure 11, Figure 12 and Figure 13.

## 4. Discussion

As demonstrated at different points in the previous chapter, the proposed IG design algorithm proved its reliability in proposing the best locations for where to inject the polymer in order to maximize the fibers’ reinforcement in specific locations of the part. To this aim, the definition of the RoI plays a pivotal role in the performance of the proposed methodology. In this research, the RoIs were chosen according to the locations of maximum equivalent stress defined through preliminary FEM simulation implemented with isotropic material properties. The RoI can also be chosen according to the user’s experience, but the definition of a preliminary FEM result allows for a better understanding of the results of phase (3), Figure 5. As important as the RoI selection, the definition of the grid size for the IG location in the FVM simulations database, phase (1), is essentially the highest influencing parameter for the computational time involved. The grid size employed in this research (15 mm) is the result of progressive iterations aimed at defining the best compromise between results accuracy and computational effort and might also be a fairly good starting point for other components. On the other hand, if the complexity or size of the investigated subject differs from those analyzed in this research, a preliminary phase considering a relatively rough grid size is suggested to avoid excessive computational effort.

As concerns the performances of the algorithm, some key elements ought to be pointed out. First, when a multiple RoI optimization is attempted, the need for a compromise naturally arises. In fact, the best IG configuration for a specific RoI might not be the same for another, and vice-versa. Thus, assigning different weights to each RoI might be of help to choose the IG configuration that allows for the prioritization of the most critical regions first. In addition, a limitation might reside in the choice of an RoI that is representative of a position in the model where the stress field is close to a hydrostatic state. In this condition, the optimization is, in fact, counterintuitive and represented by a random FOD state, since a strong alignment with only one direction might result in a weakening of the material towards the matrix-dominated directions. To avoid this issue, the proposed algorithm is based on the scores system, in turn, based on the isotropic FEM simulation, specifically aimed at identifying the key directions of the load path. This is clearly the case of the industrial pump housing, where RoI#1, close to the load application area, has a stiffness improvement between the best and worst IG far lower than the other two RoIs, located on ribs, thus with a higher directionality of the load path. Regardless of this fact, the IG design algorithm performed well under both circumstances, proving the efficacy of the scores-based system from more uniaxial to close to hydrostatic stress states.

Another key aspect to be highlighted is the difference between local and global stiffness optimization. In this research, the focus has been placed on the optimization of the FOD in specific regions of the model, which differs in nature and purpose from a global stiffness optimization. In fact, as also shown in Figure 14b, optimizing the local FOD in specific regions of the model (RoI), has a negligible effect on the global stiffness of the component, but promotes a better effectiveness of the fibers’ reinforcement in selected areas (RoI). Another key point relevant to the proposed methodology is related to the increase in the equivalent stress experienced together with the increase in stiffness. This is common to most geometries and RoIs, as shown in Figure 11, Figure 12 and Figure 13. On the other hand, it must be considered that the mentioned increase in equivalent stress is caused by a higher alignment of the fibers with the load path direction, thus also in a higher strength of the material, as inferable from Figure 3. Although out of the scope of this research, the ratio between the equivalent stress and the material’s yield for the considered FOT remains approximately the same or decreases, while the stiffness of the RoI increases, benefitting from the higher directionality of the fibers with the load path. In addition to that, since the geometry of the component, the load, and boundary conditions are fixed, the global stress state in the part cannot be altered but its redistribution within the component can be controlled by means of the FOD, which is indeed the aim of the proposed algorithm.

Although the proposed algorithm offers a viable solution to effectively design the FOD in specific regions of the part, future work should address the complex tradeoff between local and global stiffness optimization. As also demonstrated in this research, local stiffness optimization has little to no effect on the global rigidity of the component. However, in some applications, rather than local stiffness optimization, global stiffness optimization might be the target. In addition to that, the proposed approach still requires the definition of a component-based FVM database, for both end tasks of phase (3), as seen in Figure 5. Thus, more work should be focused on a general-purpose tool capable of identifying the main geometrical features of the component, already available in a trained database, and providing a close to real-time solution for the best IG design that allows for the optimization of the FOD. Finally, although the current work is based on the commonly employed stiffness design approach, the addition of strength as one of the evaluation criteria would significantly increase the applicability and usefulness of the proposed methodology.

## 5. Conclusions

This research presented an innovative methodology for the design of the injection gate location of components manufactured by short fibers-reinforced polymer in the thermoplastic injection molding process. The methodology was tested and validated against three geometries of increasing complexity and showed a substantial increase in the local stiffness, quantified in an average of 26.9%. Furthermore, when compared to experimental results on a real component, currently manufactured by the TIP process, the developed IG design algorithm showed an average local stiffness improvement of 16.4% in comparison to the currently employed IG solution. All in all, the proposed methodology proved to be effective in optimizing the local FOD in the user’s defined regions of interest within the component. This approach, combined with the very effective FVM-FEM simulation framework, allows for accounting for the influence of the IG location/s on the elastic performances of the component being designed and, for this reason, might be of help to process engineers dealing with TIM tool design and design optimization.

## Figures and Tables

**Table 1:** Material constant for the Ramberg–Osgood and anisotropic Hill ’48 yield function.

Symbol [Unit]	Definition	Value
K [MPa]	Strength coefficient	60.81
n [−]	Hardening exponent	15.97
$E_{m}\;[\mathrm{GPa}]$	Polymer matrix elastic modulus	2.21
$E_{f}\;[\mathrm{GPa}]$	Fibers’ elastic modulus	36.52
$\alpha_{m}\;[-]$	Weight factor for the fiber direction	1.42
$\beta_{m}\;[-]$	Weight factor for the direction normal to the fibers	1.17
$\lambda_{m,I}\;[-]$	First eigenvalue of the fiber orientation matrix in the region of the model with the highest fiber alignment with the polymer flow	0.85

**Table 2:** Autodesk Moldflow Insight FVM simulations process settings for injection molded plates and training geometries.

Parameter	Injection Molded Plate	Blind Hole Cylinder	Multi Features Shape	Industrial Pump Housing
Molten material temperature	290 °C	285 °C		285 °C
Mold temperature	85 °C	110 °C		110 °C
Injection time	1.51 s	Automatic		Automatic
Velocity/pressure switch-over	98.7%	99%		98%
Packing–cooling time	10 s	20 s		30 s
Post-pressure	80%	80%		85%

**Table 3:** Number of FVM simulations for the three components (IG and FOT DB of Phase 2).

Component	IG = 1	IG = 2	IG = 3
Blind hole cylinder	90	90	90
Multi features shape	110	110	110
Industrial pump housing	160	160	160

**Table 4:** Optimized hyperparameters for the three geometries and average accuracies for the training and validation k-fold (k = 5) process.

Parameter	Blind Hole Cylinder	Multi Features Shape	Industrial Pump Housing
Max depth	10	9	9
Min samples leaf	7	7	7
Min samples split	8	6	5
Estimators number (*M*)	100	100	100
Learning rate (*η*)	0.1	0.1	0.1
Loss function (*δ*)	0.9	0.9	0.9
Training accuracy (5-fold average)	94%	97.7%	98.1%
Validation accuracy (5-fold average)	91.4%	97.4%	97.8%

**Table 5:** Optimized hyperparameters for the three geometries and average accuracies for the training and validation k-fold (k = 5) process.

IG Coordinates		Blind Hole Cylinder			Multi Features Shape			Industrial Pump Housing		
		x	y	z	x	y	z	x	y	z
Pre GB-ML	IG = 2 (#1)	76.8	76.8	72.0	13.7	28.0	41.6	10.45	13.96	36.8
	IG = 2 (#2)	13.8	76.8	87	60	10	60	22.7	25.2	57.2
Post GB-ML	IG = 2 (#1)	73.9	79.5	67.4	63.8	10	56	15.5	18.4	45.8
	IG = 2 (#2)	60.6	79.7	0	12.9	31.6	41.8	24.8	25.2	36.0
Average ROI Stiffness improvement		8.6%			5.1%			6.3%		

## Data Availability

All raw data and algorithms are available on the https://1drv.ms/f/s!Am_ASYI2QBIriOlnSmrkxhQg7vcJGA?e=bEVxfx (accessed on 30 May 2023) online repository on can be requested to the corresponding author.
